# Ultrathin, flexible and multimodal tactile sensors based on organic field-effect transistors

**DOI:** 10.1038/s41598-018-26263-1

**Published:** 2018-05-23

**Authors:** Fabrizio Antonio Viola, Andrea Spanu, Pier Carlo Ricci, Annalisa Bonfiglio, Piero Cosseddu

**Affiliations:** 1 Department of Electrical and Electronic Engineering, University of Cagliari, Piazza d’Armi, 09123 Cagliari, Italy; 2 Micro-Systems Technology Group, Fondazione Bruno Kessler, Trento, 38123 Italy; 3 Department of Physics, University of Cagliari, S. P. 8, I-09042, Monserrato, Cagliari, Italy

## Abstract

In this study, a novel approach to the fabrication of a multimodal temperature and force sensor on ultrathin, conformable and flexible substrates is presented. This process involves coupling a charge-modulated organic field-effect transistor (OCMFET) with a pyro/piezoelectric element, namely a commercial film of poly-vinylene difluoride (PVDF). The proposed device is able to respond to both pressure stimuli and temperature variations, demonstrating the feasibility of the approach for the development of low-cost, highly sensitive and conformable multimodal sensors. The overall thickness of the device is 1.2 μm, being thus able to conform to any surface (including the human body), while keeping its electrical performance. Furthermore, it is possible to discriminate between simultaneously applied temperature and pressure stimuli by coupling sensing surfaces made of poled and unpoled spin-coated PVDF-trifluoroethylene (PVDF-TrFE, a PVDF copolymer) with OCMFETs. This demonstrates the possibility of creating multimodal sensors that can be employed for applications in several fields, ranging from robotics to wearable electronics.

## Introduction

The increasing ubiquity of portable and wearable technologies has resulted in an increasing interest in flexible, conformable and lightweight electronic devices. Since the discovery of conductive polymers^1^, a significant amount of research effort has been devoted to devices that can be conformed to different substrates, resulting in the development of new techniques for the fabrication of flexible photovoltaic modules^2^, displays^3^ and printed circuits^4,5^. Furthermore, the recent rise of the so-called “tattoo electronics” has pushed further the innovation on flexible and ultra-conformable electronic devices that can be transferred onto the skin, particularly meant for biomedical applications.

Organic electronics represents the natural candidate for the fulfillment of the increasing needs of this emerging application field, since the employed materials are usually biocompatible, transparent and intrinsically flexible. Furthermore, within this field innovative fabrication techniques offer unprecedented versatility, allowing the realization of a broad range of different devices onto large areas and at low cost^6^.

An interesting example of this new trend is represented by the work of John Roger’s group, who has investigated materials that are able to replicate the mechanical properties of the skin by proposing a novel approach towards the development of a variety of conformable “epidermal electronic systems”. These systems are specifically engineered in order to adhere to the skin and they include several kinds of electronic devices, such as sensors, light-emitting diodes, photodetectors^7^.

Other interesting approaches have been proposed to design ultra-flexible electronics for biomedical applications, such as the one proposed by Campana *et al*.^8^, who presented a conformable organic electrochemical transistor (OECT) on a fully resorbable bioscaffold manufactured using poly (L-lactide-co-glycolide) for recording electrocardiographic (ECG) data. Kaltenbrunner *et al*.^9^ introduced an ultra-flexible and extremely stress-resilient electronic circuit fabricated on a very thin polyethylene naphthalate sheet (1.2 µm). Recently, Lai *et al*. successfully fabricated complementary inverters and single-stage amplifiers with a sub-micrometer thickness on polymer nanosheets for epidermal electronics applications^10^. Using a different approach, Someya’s group fabricated tactile sensing films as thin as 300 nm on Parylene C substrates that can be transferred to human skin without causing dermal irritation^11^.

Beside the application of organic sensors in bio-electronics and wearable electronics, an increasing effort has been focused on the development of novel approaches for the fabrication of an “electronic skin” that can replicate the sense of touch^12–20^ for prosthetics applications, human-robot interactions and rehabilitation. In particular, tactile systems have been investigated as tools to transduce information about an object (such as shape, temperature, superficial texture, etc.) through physical interaction. An ideal tactile sensor should be capable of simulating the sensing behavior of the human skin in terms of the spatial resolution and sensitivity and dynamic range for sensing force and temperature^18^.

Despite the various approaches that have been employed so far^12–20^, fabricating tactile sensors on ultra-conformable substrates remains a challenge. Most of the devices that have been previously developed for pressure and temperature transduction for tactile applications are based on coupled piezoresistive/pyroresistive devices^21–25^ or the combination of piezoelectric and pyroelectric materials^26,27^. Although these electronic devices do offer flexibility, they are fabricated using materials that have thickness values of several microns or high elastic moduli, therefore failing to provide conformability, which is necessary for epidermal applications.

Another important requirement for e-skin applications is the possibility of simultaneously transducing different types of stimuli with high reliability. To this aim, Graz *et al*.^28^ developed a bifunctional device for temperature and pressure detection using two integrated piezoelectric/pyroelectric sensing elements based on a composite foil of poly-vinylene difluoride-trifluoroethylene (PVDF-TrFE, a ferroelectric material) and a piezoelectric ceramic coupled to a transistor structure. Lee *et al*.^29^ depicted a highly sensitive multifunctional tactile sensor based on PVDF and ZnO nanostructures with graphene electrodes. The main advantages of using piezoelectric/pyroelectric sensing elements rely on their good high frequency responses and their high sensitivities^30^. Very recently, Zhao and Zhu developed an electronic skin with multifunctional sensors based on a pair of platinum (Pt) variable resistances integrated in a Wheatstone bridge. This system is able to simultaneously detect temperature and pressure variations with negligible hysteresis and high sensitivity^31^.

In this study, a particular kind of organic thin-film transistor (OTFT) called organic charge-modulated field-effect transistor (OCMFET) is coupled to different piezoelectric/pyroelectric polymers, such as PVDF and PVDF-TrFE, to obtain ultra-thin multimodal force and temperature sensors. During the last ten years, the OCMFET has been tested for several sensing and bio-sensing applications including pH sensing^32,33^, DNA hybridization sensing^34^, force transduction^35^ and electrogenic-cells activity detection^36^. The device proposed in this study offers significant advantages over the previously reported systems:

1. The overall thickness of the device is 1.2 µm, which allows it to meet the above-mentioned conformability requirement.
2. In contrast to other FET-based approaches, the sensing and the amplification elements are here physically separated; this prevents the external stimulus (temperature and/or force) from affecting the intrinsic characteristics of the semiconductor.
3. The device itself is based on a single sensitive material (PVDF), which has both piezoelectric and pyroelectric properties^37^. This allows a single fabrication process to be used for both temperature and pressure sensing.
4. The signal transduction mechanism does not strictly depend on the specific semiconductor in use. Therefore, this design choice gives the possibility of developing devices based on a similar concept with different fabrication technologies using not only organic materials but also inorganic or hybrid materials.

We here describe the optimization process of the layout that has been made on such a peculiar transducer in order to obtain the best performance in terms of sensitivity. We also provide a complete mechanical and thermal characterization of the sensor, with the intent of demonstrating the suitability of the proposed approach for e-skin applications.

## Results

**Figure 1:**
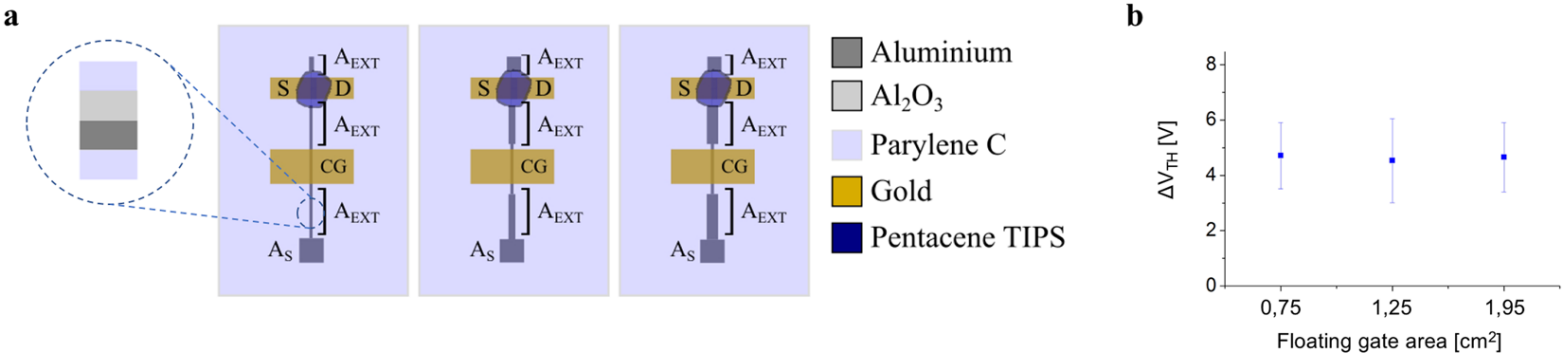
OCMFET layouts and V_TH_ dependence on the floating gate area. (**a**) Schematic representation of the three different OCMFET structures that have been tested to assess the dependence of the V_TH_ from the external areas (5 devices for each external area dimension). The transistor and the control gate area are the same in all three devices. (**b**) Shift of the threshold voltage (ΔV_TH_) after the connection of the floating gate with the PVDF. No correlation was observed between ΔV_TH_ and the floating gate area.

The OCMFET is a floating-gate OTFT in a bottom-gate/bottom-contact configuration that is biased using a control capacitor. As shown in Fig. 1, a key feature of the OCMFET is represented by its elongated floating gate such that the sensing area (i.e. the part of the floating gate in which the actual transduction happens) is far from the transistor area. The mechanism of OCMFET operation is related to a variation of the transistor threshold voltage V_TH_ that is induced by a charge variation (ΔQ_S_) occurring on the sensing area, which in turn causes a charge variation (ΔQ_T_) under the transistor region. It is possible to derive the relationship between the control gate voltage V_CG_, the induced charge Q_i_ and the transistor’s effective threshold voltage V_THF_ starting from Gauss’ law:

1 $${{\rm{Q}}}_{{\rm{f}}}={{\rm{C}}}_{{\rm{CG}}}({{\rm{V}}}_{{\rm{FG}}}-{{\rm{V}}}_{{\rm{CG}}})+{{\rm{C}}}_{{\rm{SF}}}{{\rm{V}}}_{{\rm{FG}}}+{{\rm{C}}}_{{\rm{DF}}}({{\rm{V}}}_{{\rm{FG}}}-{{\rm{V}}}_{{\rm{D}}})+{{\rm{Q}}}_{{\rm{i}}},$$

where Q_f_ is the total charge in the floating gate, V_FG_ is the floating gate voltage, V_D_ is the drain voltage, C_CG_ is the capacitance of the control capacitor, C_SF_ and C_DF_ are the parasitic capacitors between the floating gate and the source and the drain electrodes. Is then possible to express the floating gate voltage as follows:

2 $${{\rm{V}}}_{{\rm{FG}}}=\frac{({{\rm{Q}}}_{{\rm{f}}}-{{\rm{Q}}}_{{\rm{i}}})}{{{\rm{C}}}_{{\rm{TOT}}}}+\,\frac{{{\rm{C}}}_{{\rm{CG}}}}{{{\rm{C}}}_{{\rm{TOT}}}}{{\rm{V}}}_{{\rm{CG}}}+\frac{{{\rm{C}}}_{{\rm{DF}}}}{{{\rm{C}}}_{{\rm{TOT}}}}{{\rm{V}}}_{{\rm{D}}}.$$

The effective threshold voltage is now easily derivable from the following set of equations:

3 $${{\rm{V}}}_{{\rm{CG}}}-{{\rm{V}}}_{{\rm{THF}}}={{\rm{V}}}_{{\rm{FG}}}-{{\rm{V}}}_{{\rm{TH}}}\,$$

4 $${{\rm{V}}}_{{\rm{CG}}}-{{\rm{V}}}_{{\rm{THF}}}={{\rm{V}}}_{{\rm{FG}}}-{{\rm{V}}}_{{\rm{TH}}}=\frac{({{\rm{Q}}}_{{\rm{f}}}-{{\rm{Q}}}_{{\rm{i}}})}{{{\rm{C}}}_{{\rm{TOT}}}}+\,\frac{{{\rm{C}}}_{{\rm{CG}}}}{{{\rm{C}}}_{{\rm{TOT}}}}{{\rm{V}}}_{{\rm{CG}}}+\frac{{{\rm{C}}}_{{\rm{DF}}}}{{{\rm{C}}}_{{\rm{TOT}}}}{{\rm{V}}}_{{\rm{D}}}-{{\rm{V}}}_{{\rm{TH}}}.$$

5 $${{\rm{V}}}_{{\rm{THF}}}\cong {{\rm{V}}}_{{\rm{TH}}}-\frac{{{\rm{C}}}_{{\rm{DF}}}}{{{\rm{C}}}_{{\rm{TOT}}}}{{\rm{V}}}_{{\rm{D}}}-\frac{({{\rm{Q}}}_{{\rm{f}}}-{{\rm{Q}}}_{{\rm{i}}})}{{{\rm{C}}}_{{\rm{TOT}}}}.$$

where C_CG_/C_TOT_ $\cong$ 1 (the parasitic capacitances are in fact negligible with respect to C_CG_). If all the terms are constant, but the charge Q_i_ change, the threshold voltage shift can be written as:

6 $${{\rm{\Delta }}{\rm{V}}}_{{\rm{TH}}}=-\frac{{\rm{\Delta }}{{\rm{Q}}}_{i}}{{{\rm{C}}}_{{\rm{TOT}}}}.$$

One of the main advantages that is offered by the use of this structure (especially with respect to the passive thermal resistors) is that the charge induced on the sensing area is locally amplified by the transistor itself. Furthermore, the control gate can be used to minimize the spread of the electrical parameters (i.e. the threshold voltage and mobility) due to the fabrication process by impose the same over-threshold condition to all the devices in an array configuration.

As a preliminary test, the influence of the floating gate layout on the sensor response was examined to properly design the sensor architecture for a specific application. As extensively explained in a recent study from this group^33^, the charge in the sensing area (A_S_) determines a superficial charge variations (Δσ) in the transistor area (A_T_), the control gate area (A_CG_) and the remaining parts of the floating gate (collectively indicated as A_EXT_), according to the following expression:

7 $${{\rm{\Delta }}{\rm{Q}}}_{{\rm{S}}}={{\rm{\Delta }}{\rm{\sigma }}}_{{\rm{S}}}{{\rm{A}}}_{{\rm{S}}}={{\rm{\Delta }}{\rm{\sigma }}}_{{\rm{T}}}{{\rm{A}}}_{{\rm{T}}}+{{\rm{\Delta }}{\rm{\sigma }}}_{{\rm{EXT}}}{{\rm{A}}}_{{\rm{EXT}}}+{{\rm{\Delta }}{\rm{\sigma }}}_{{\rm{CG}}}{{\rm{A}}}_{{\rm{CG}}}.$$

To investigate the hypothesis that only a negligible charge redistribution occurs in the external areas, three different OCMFET structures having the same transistor, sensing and control-gate areas but different dimensions of the remaining floating-gate areas (A_EXT_) were fabricated, as depicted in Fig. 1. In Table S1 in Supplementary Materials is reported a complete description of the floating gate, control gate, transistor and sensing areas for each type of OCMFET structure. The number of devices tested is higher than 5 for each layout. By coupling one plate of the PVDF capacitor to the sensing area, a considerable charge is induced in the floating gate due to the intrinsic polarization of PVDF. This modulates the concentration of charge carriers in the OFET channel, which alters its threshold voltage. The V_TH_ variation was evaluated for the three different A_EXT_ before and after connecting them to the PVDF capacitor. As illustrated in Fig. 1, no correlation was observed between ΔV_TH_ and the size of A_EXT_ within the range tested. In such a situation, Equation 2 can be simplified by considering the charge under the control gate to be fixed, as follows:

8 $${{\rm{\Delta }}Q}_{{\rm{S}}}={{\rm{\Delta }}{\rm{\sigma }}}_{{\rm{S}}}{{\rm{A}}}_{{\rm{S}}}={{\rm{\Delta }}{\rm{\sigma }}}_{{\rm{T}}}{{\rm{A}}}_{{\rm{T}}}.$$

This experimentally confirms the hypothesis in^33^.

Following this preliminary evaluation, the device was implemented as a multimodal temperature and force/pressure sensor based on the following principle: when a temperature or force variation occurs on the PVDF film, a charge separation is induced across the film due to the pyroelectric/piezoelectric properties of the material. This charge, depending on its sign, influences the carrier density within the transistor channel, thus causing a modulation of the transistor’s field effect. According to this mechanism, the threshold voltage shifts in response to a thermal or mechanical stress on the capacitor due to the piro-/piezoelectric property of PVDF, resulting in a modulation of the output current of the OCMFET and I_DS_.

**Figure 2:**
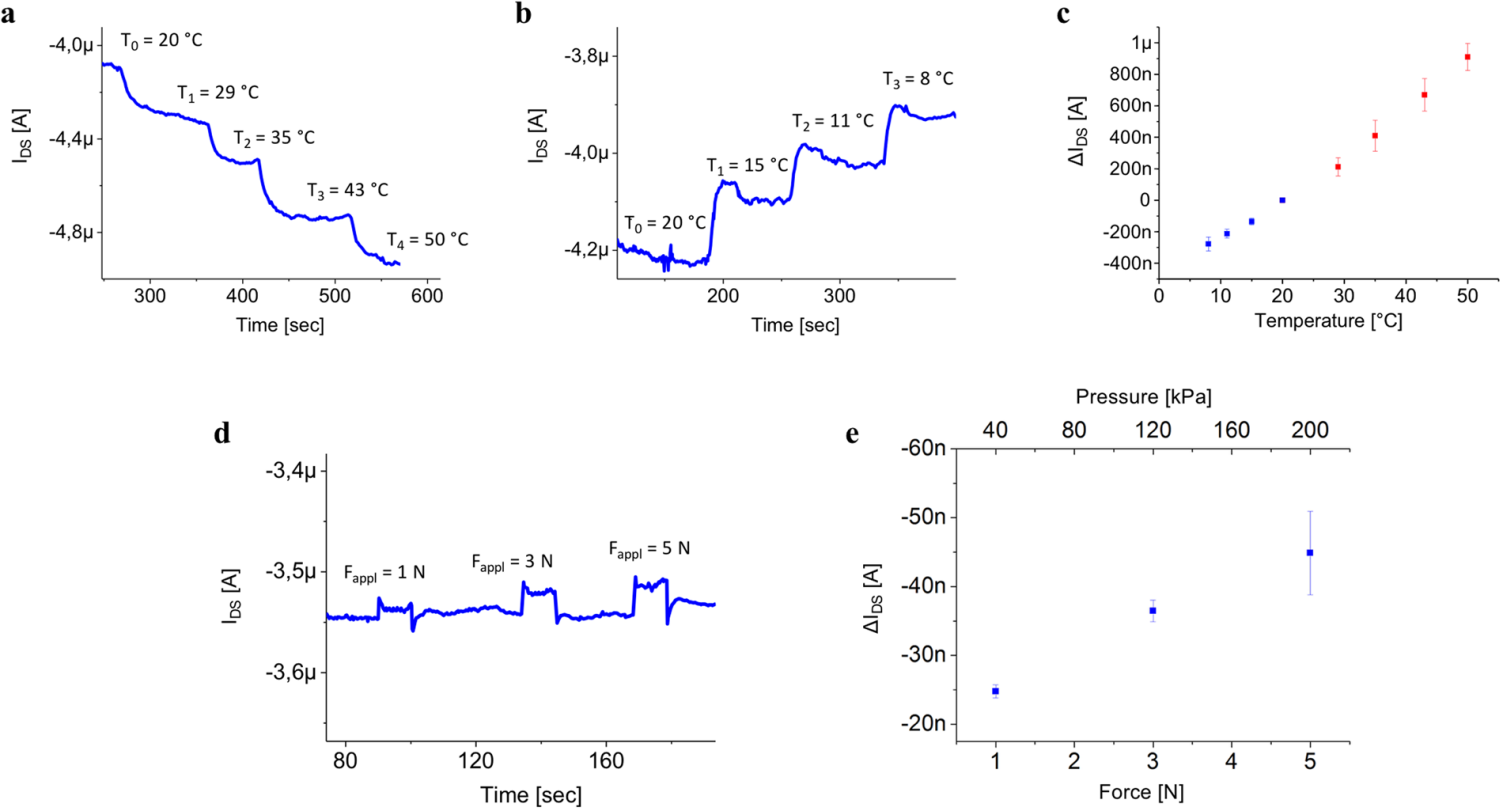
OCMFET electrothermal and electromechanical characterization. (**a–b**) Variations in the OCMFET current (I_DS_) upon the application of a thermal stress to the PVDF capacitor coupled to the floating gate. (**c**) Calibration curves of the sensor, blue (red) points represent the variations of the output current for temperature decrease (increase). The device exhibited a reproducible linear response in the range 8 °C–50 °C. (**d**) Variations in I_DS_ upon the application of mechanical stress to the PVDF capacitor coupled to the floating gate. (**e**) Electromechanical characterization of the sensor.

The devices have been initially characterised separately as force and temperature sensors. The temperature and force variations were induced on the PVDF film using a Peltier cell and a mechanical indenter (IMADA Digital Force Gauge) respectively. For this characterisation we tested 10 devices, and for each device we performed 5 cycles of temperature/force measurements. All the devices used in our experiments were operated with V_DS_ = V_GS_ = −2V. As explained in Fig. 2a,b and c, the electro-thermal characterization has been performed by gradually increasing (or decreasing) the temperature while I_DS_ was monitored. Each temperature step induces a corresponding change in I_DS_, which eventually reaches a steady-state when the temperature stabilizes. The results of this characterization demonstrate that the proposed approach can be used for temperature monitoring within the range 8 °C–50 °C, which is the temperature range that is required for most tactile applications^38^. Interestingly, as reported in the graph in Fig. 2(c), we observed a clear and reproducible linear response to the applied thermal stimuli. Such an approach turns out to be feasible also for mechanical sensing, as shown in Fig. 2e,f where the electromechanical characterization of the sensor is presented. The applied force was precisely measured using a dynamometer to obtain a reliable calibration curve. The results depict that the sensor is able to detect applied forces in the range of 1–5 N (corresponding to 40 kPa–200 kPa), which is consistent with the required target range for tactile applications. Again, the obtained response turned out to be linear and highly reproducible.

**Figure 3:**
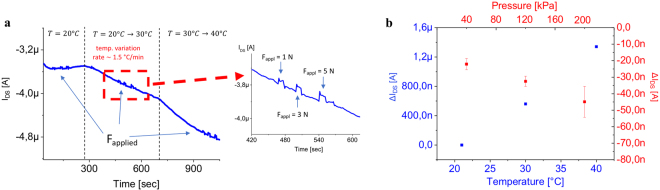
Simultaneous OCMFET response to force and temperature stimuli. (**a**) Dynamic response of the sensor to the simultaneous application of temperature and force stimuli to the PVDF. (**b**) Characterisation of the temperature and force sensitivities.

**Figure 4:**
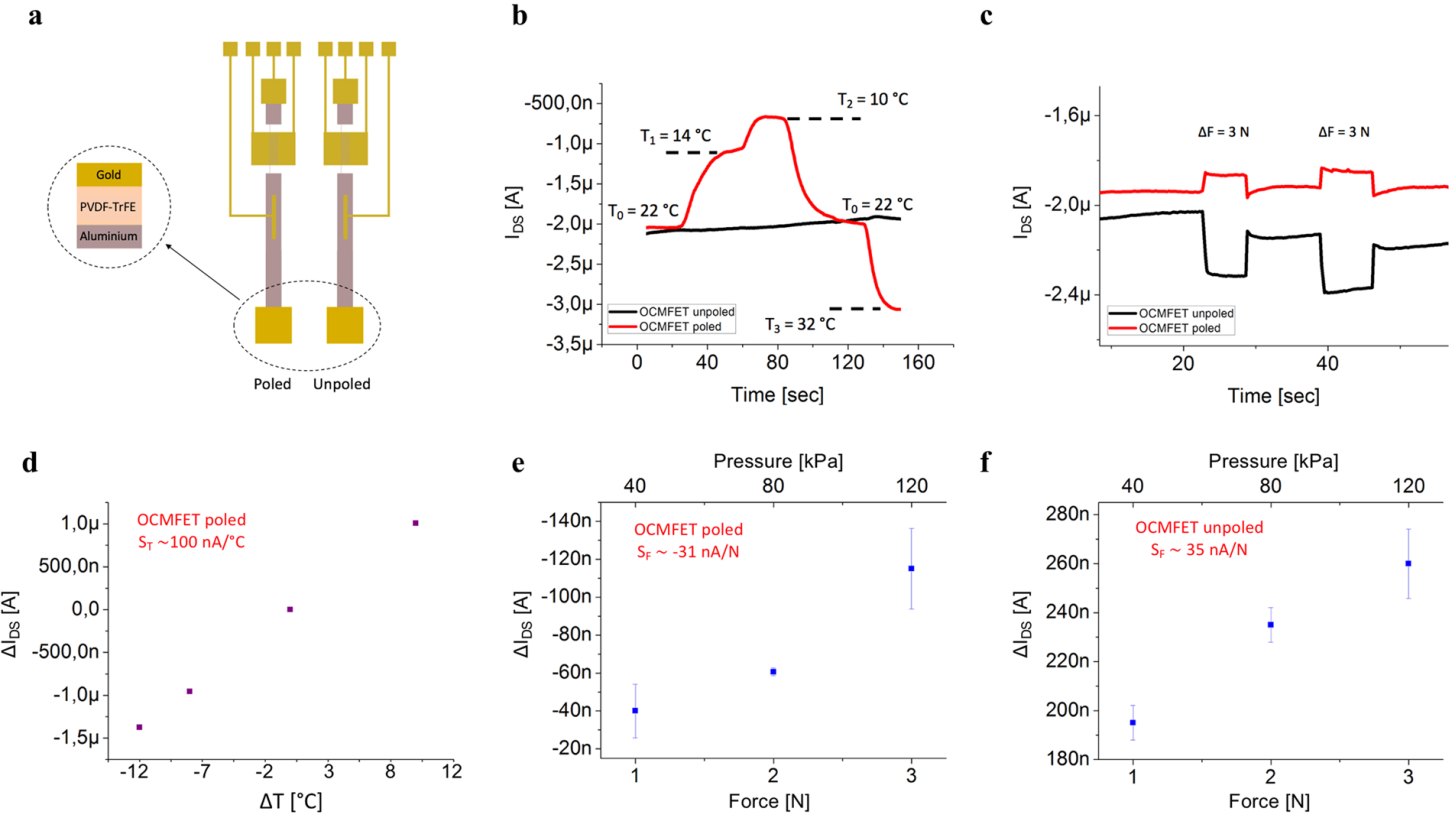
OCMFET pixel for bimodal sensing. (**a**) Schematic representation of OCMFETs with PVDF-TrFE spin-coated onto the sensing area. (**b**) I_DS_ variations upon the application of a thermal stimulus to the sensing area with poled PVDF-TrFE (red curve) and unpoled PVDF-TrFE (black curve). (**c**) Dynamic response of the sensor to the application of force stimuli to the sensing elements. (**d**) Calibration curve and sensitivity of the poled OCMFET in response to thermal stimuli. (**e**) Calibration curve and sensitivity of the poled OCMFETs in response to mechanical stimuli. (**f**) Calibration curve and sensitivity of the unpoled OCMFETs in response to mechanical stimuli.

With the intent of proving that the proposed approach can be actually employed for practical applications, the capability of the sensor to discriminate between temperature and force variations that were applied simultaneously was also investigated. The sensing area of the OCMFET was placed on a Peltier cell that was fixed to the sample holder of a mechanical indenter. In this way, the temperature of the sensing area is monitored while a measurable force can be simultaneously exerted on the same area (Fig. 3). Initially, the sensing area was kept at a constant temperature (T = 22 °C) while being mechanically stimulated using a force of either 1, 3 or 5 N. As expected, the value of I_DS_ varies proportionally with the magnitude of the applied force. The temperature of the sensing area was then linearly increased (with a temperature variation rate $\sim$ 1.5 °C/min) from 22 °C to 50 °C, causing a linear decrease of the output current, while the mechanical stimulus was applied (1, 3 or 5 N). The results illustrated in Fig. 3a shows that the sensor is able to respond to applied pressures simultaneously during thermal stimulation with good reproducibility (see inset). As depicted in Fig. 3b, the sensitivities to thermal and mechanical stimuli (S_T_ and S_F_, respective), defined as

9 $${{\rm{S}}}_{{\rm{T}}}=\frac{{\rm{\partial }}{{\rm{I}}}_{{\rm{D}}{\rm{S}}}}{{\rm{\partial }}{\rm{T}}}\,{\rm{a}}{\rm{n}}{\rm{d}}\,{{\rm{S}}}_{{\rm{F}}}=\frac{{\rm{\partial }}{{\rm{I}}}_{{\rm{D}}{\rm{S}}}}{{\rm{\partial }}{\rm{F}}},$$

lie in very different ranges, namely 70 nA/°C and 6 nA/N (corresponding to 0.15 nA/kPa), respectively. Moreover, the response to an applied force does not appear to be significantly affected by the temperature of the sensing area. Interestingly, considering the typical force range that is required for tactile applications (0.01–5 N)^39^, it can be observed that a temperature variation as low as 1 °C elicits a higher current variation than a pressure stimulus of 10 N, thus making, in realistic tactile applications, the two responses easily distinguishable by employing a simple signal processing routine. However, in order to be able to simultaneously record and distinguish pressure and temperature without any previous knowledge on the magnitude of the stimuli, we propose an alternative approach consisting of building a “pixel” containing two OCMFETs: one with a poled PVDF-TrFE layer spin-coated onto the sensing area and one with the same spin-coated PVDF-TrFE layer but unpoled (Fig. 4a). The reasons behind this solution stem from the different behavior of the same material before and after the poling procedure and the choice of using the copolymer PVDF-TrFE allows the use of an easier poling procedure compared to what is required for standard PDVF^40^. Moreover, it has been demonstrated that spin coated PVDF-TrFE thin films show a high β-phase formation without electrical poling, which correspond to high piezoelectricity^41^. In fact, even without the poling step, PVDF-TrFE samples present a residual polarization which increases as the annealing time increases^42^. As reported in Fig. S3 in Supplementary Materials, Raman spectra of our PVDF-TrFE unpoled samples annealed for 120 minutes @60 °C, @100 °C and @140 °C, show the presence of an evident reduction of paraelectric alpha-phase—peak at about 800 cm^−1 ^^43^—and an increase of a piezoelectric beta-phase—peak at 840 cm^−1 ^^41^—with the increase of the annealing temperature. On the other hand, it has been demonstrated that PVDF-TrFE pyroelectricity decades with the increase of annealing temperature^44,45^. As depicted in Fig. 4b,c, unpoled PVDF-TrFE is insensitive to temperature stimuli but retains its sensitivity to mechanical stimuli due to the intrinsic piezoelectricity of the sensing element. By fabricating a pixel structure containing two devices—one that is only sensitive to pressure and the other that is sensitive to both pressure and temperature—it is possible to discriminate between the two stimuli by evaluating the difference between the two signals. The calibration curves and sensitivities of an OCMFET with the PVDF-TrFE layer deposited onto the sensing area are showed in Fig. 4d–f. Similar to the previous tests, the devices were able to sense temperatures and forces in the ranges that are relevant to tactile applications, also showing, as expected, different sensitivities to thermal and force stimuli. Furthermore, it is in principle possible to discriminate between the two stimuli, regardless of their dynamics using a simple pixel configuration. The most significant advantage of this approach is that this multi sensing device can be fabricated using only one kind of electronic device and material, making the system very simple, convenient and practical for use in epidermal applications.

**Figure 5:**
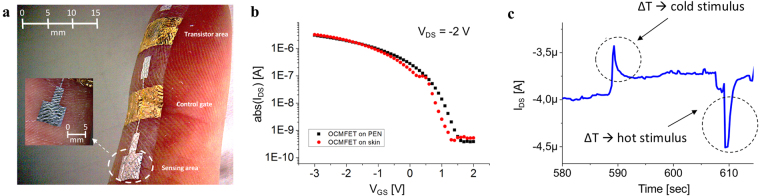
OCMFET electrical characterization on skin. (**a**) OCMFET placed on the skin, demonstrating its good conformability. (**b**) Electrical characterisation of the device before being peeled-off from the PEN carrier and after its placement onto the skin. (**c**) Preliminary results of the temperature sensing on skin.

Finally, to demonstrate the suitability of the proposed sensor architecture for tactile applications, the sensor was transferred onto a volunteer subject’s skin and its response to a thermal stimulus was measured. Figure 5a depicts that the fabricated sensor structure can be conformably transferred onto human skin due to its overall micrometer-scale thickness, and shows that such devices can be positioned onto a fingertip with a high level of conformability and adherence to the skin.

Moreover, the intrinsic electrical characteristics of the OCMFET (without the sensing element connected to the floating gate) do not change upon transferring it to the skin, as depicted in Fig. 5b. In addition, as showed in Fig. S4 in Supplementary Materials, the device can be bent with very small bending radii (lower than 0.02 cm) with no significant variations in the OCMFET electrical characteristics, even after more than 200 cycles. After coupling the PVDF to the sensing area, a qualitative test was performed to evaluate the capability of the sensor to detect temperature variations. In particular, either a warm or cold object was placed in proximity of the sensing area while monitoring the sensor’s output. As shown in Fig. 5c, the results demonstrate that I_DS_ increases when a warm stimulus is brought near the sensor, and, conversely, decreases when the cold object is presented to the sensing area instead. This qualitative experiment, though preliminary, demonstrates that the intrinsic electrical performance of the transistor is preserved (and the sensing capabilities of the OCMFET are maintained) after transferring the sensing system directly onto the human skin.

## Discussion

In conclusion, a multimodal device for both temperature and force sensing has been developed. The sensor can be conformably transferred onto various surfaces, such as the human skin, due to its micrometer-scale thickness with no significant changes in the properties of the device. Complete thermal and electromechanical characterizations of the sensor in the typical ranges that are required for tactile applications (8 °C–50 °C and 1–5 N, respectively) were successfully conducted. Moreover, an easy and convenient approach for the simultaneous detection of the two stimuli has been proposed, thus demonstrating the possibility of using such a PVDF-TrFE/OCMFET system for multimodal tactile applications such as e-skin and epidermal electronics.

## Methods

To fabricate the sensors, poly ethylene naphtalate (PEN - Goodfellow) was used as a carrier substrate. A soap solution (2 wt.% in water) was spin-coated onto the carrier substrate in order to reduce the adhesion of the subsequently deposited Parylene C layer to the carrier substrate, thus facilitating its removal at the end of the fabrication process. Parylene C, which was chosen as the substrate for the micro-scale transistors, was then deposited on the carrier substrate by chemical vapour deposition using a Labcoater 2 SCS PDS 2010 (Specialty Coating System) to obtain an, ultra-thin (thickness ≅ 900 nm) substrate. The core of the sensing system is an aluminum floating gate electrode, which is deposited and patterned using standard photolithography. The dielectric layer comprises a 6 nm-thick Al2O3 layer that was developed by thermal treatment and 150 nm-thick Parylene C film. After the dielectric layer deposition, gold source and drain electrodes were patterned on the Parylene C film using the same standard photolithographic process used for the floating gate fabrication. For the experimental session described in the first part of the manuscript, a 28 μm-thick poled PVDF film (Measurement Specialties Inc., MEAS; area = 5 × 5 mm^2^) was glued to the sensing area of the transistor using a conductive silver paste.

For the PVDF-TrFE experiments, a solution of 10 wt.% of 70/30 PVDF-TrFE (Piezotech, ARKEMA Group) was dissolved in DMSO and spin-coated onto the OCMFET sensing area. The solvent was evaporated at 140 °C for 2 h, obtaining a PVDF-TrFE layer with a final thickness of approximately 3 μm. Gold top electrodes were evaporated and patterned onto the PVDF-TrFE polymer using a shadow mask. The poling process was performed at 80 °C, and a voltage was applied across the polymer in incremental steps of 50 V at 10-min intervals up to 200 V. For all the devices, 6,13-Bis(triisopropylsilylethynyl)pentacene (TIPS Pentacene, Sigma-Aldrich) was used as a p-type organic semiconductor. A solution of 0.5 wt.% of TIPS Pentacene in anisole was drop-casted onto the channel area. The fabricated devices were finally released from the carrier substrate by dry peeling with the aid of an adhesive tape frame.

Raman scattering measurements were carried out in backscattering geometry using a 532.0 nm line by a wavelength stabilized diode module (LASOS DPSS series) coupled with a Reflecting Bragg Grating (Optigrate–Braggrade 405) to narrow the laser line. Measurements were performed at room temperature with a triple spectrometer Jobin-Yvon Dilor integrated system with a spectral resolution of about 1 cm^−1^. Spectra were recorded in the Stokes region by a 1200 groove/mm grating monochromator and a LNcooled charge coupled device (CCD) detector system.

The study was performed following the principles outlined in the Helsinki Declaration of 1975, later revised in 2000. All participants signed up an informed consent form before the experimental activity began, after being informed about the aims and procedures of the experiments.

## Electronic supplementary material

Supplementary Materials
