# Supplementary material for: Ultrathin, flexible and multimodal tactile sensors based on organic field-effect transistors
Source: Sci Rep. 2018 May 23;8:8073. doi: 10.1038/s41598-018-26263-1 (PMC5966445; doi:10.1038/s41598-018-26263-1)
Supplement: Supplementary file 1 — Supplementary Materials [file 41598_2018_26263_MOESM1_ESM.docx]

**SUPPLEMENTARY MATERIALS**

Ultrathin, flexible and multimodal tactile sensors based on organic field-effect transistors

**Authors:** F. Viola^1*^, A. Spanu^1,2^, P. C. Ricci^3^, A. Bonfiglio^1^ and P. Cosseddu^1^

**Affiliations:**

^1^University of Cagliari – Dept. of Electrical and Electronic Engineering, Piazza d’Armi, 09123 Cagliari, Italy

^2^ Micro-Systems Technology Group, Fondazione Bruno Kessler, Trento 38123, Italy

^3^ University of Cagliari – Dept. of Physics, S. P. 8, I-09042, Monserrato, Cagliari, Italy

*Corresponding author. Email: fabrizio.viola@diee.unica.it

**Supplementary Materials**

In Table S1 a complete description of the floating gate areas for each type of OCMFET structure is shown.

| **OCMFET** | **A_T_ [cm^2^]** | **A_CG_ [cm^2^]** | **A_S_ [cm^2^]** | **A_EXT_ [cm^2^]** | **A_FG_ [cm^2^]** |
| --- | --- | --- | --- | --- | --- |
| **Structure 1** | 1 x 10^-2^ | 2.65 x 10^-2^ | 2.5 x 10^-1^ | 4.635 x 10^-1^ | 7.5 x 10^-1^ |
| **Structure 2** | 1 x 10^-2^ | 2.65 x 10^-2^ | 2.5 x 10^-1^ | 9.635 x 10^-1^ | 12.5 x 10^-1^ |
| **Structure 3** | 1 x 10^-2^ | 2.65 x 10^-2^ | 2.5 x 10^-1^ | 16.635 x 10^-1^ | 19.5 x 10^-1^ |

Supplementary Table S1: Dimension of the different areas for each type of OCMFET employed.

In Figure S1 the complete structure of the multimodal sensor is shown, in Figure S2 are shown the transfer characteristic of three OCMFET with different structure, before and after the coupling with the PVDF capacitor.


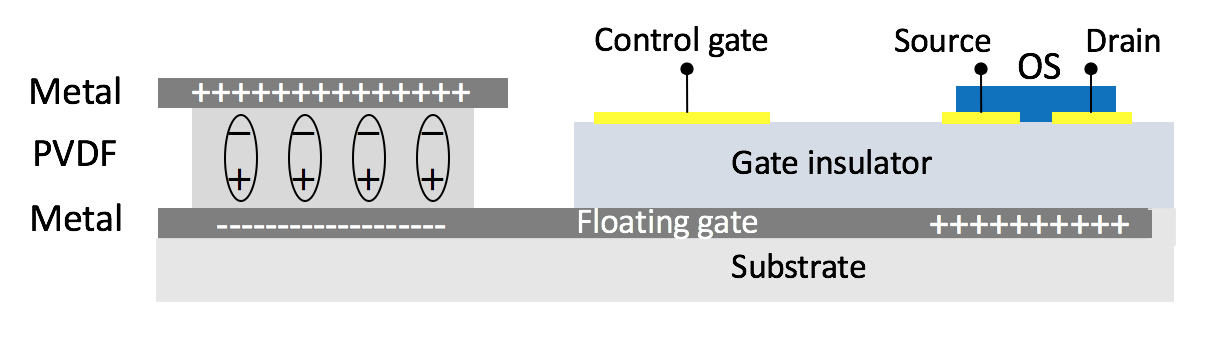


**Supplementary Fig. S1. OCMFET cross section.** Schematic illustration of an OCMFET for mechanical and thermal sensing.


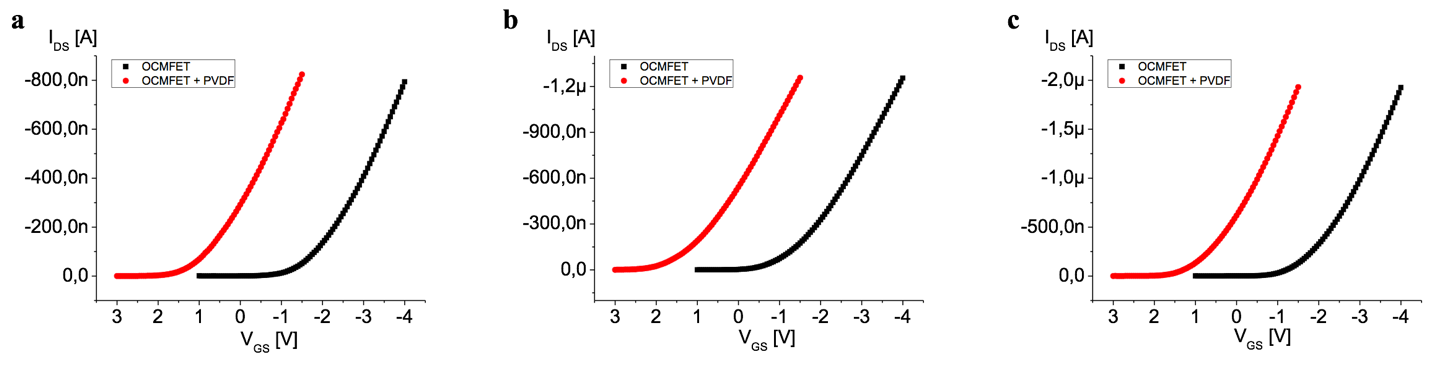


**Supplementary Fig. S2. V_TH_ variation evaluation.** Transfer characteristics of OCMFET before (black points) and after (red points) the coupling with the PVDF capacitor. In **(a)** is shown a transfer characteristic of one OCMFET with Structure 1, in **(b)** a transfer characteristic of one OCMFET with Structure 2, in **(c)** a transfer characteristic of one OCMFET with Structure 3. V_DS_ = -4 V for all the measurements.

**
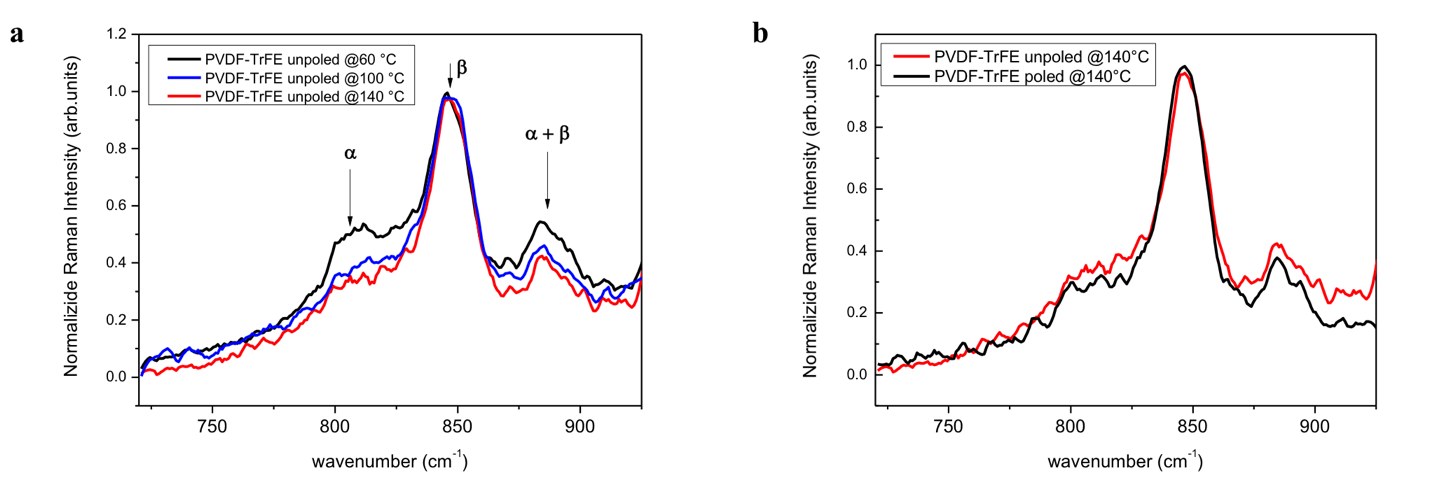
**

Supplementary Fig. S3. PVDF-TrFE Raman spectra. In (a) is shown the Raman spectra of the unpoled PVDF-TrFE samples annealed @60 °C, @100 °C and @140°C, in (b) is reported the Raman spectra of the PVDF-TrFE poled and unpoled annealed @140 °C.


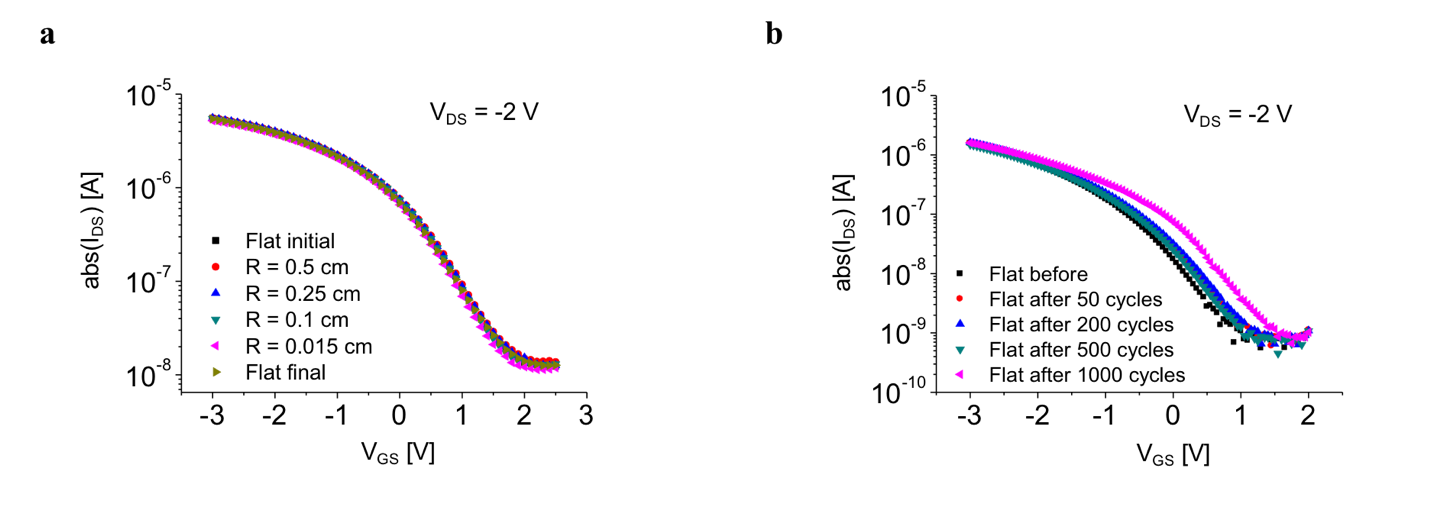


Supplementary Fig. S4. Bending tests on ultrathin OCMFET. (a) Transfer characteristics of OCMFET flat and bent at different bending radii. (b) Transfer characteristics of OCMFET before and after bending tests (bending radius = 150 μm).
